# Recent Progress in Health Benefits of Hederagenin and Its Glycosides

**DOI:** 10.3390/molecules30163393

**Published:** 2025-08-15

**Authors:** Guangjie Zhang, Yining Feng, Li Huang, Chenxi Ren, Mingyuan Gao, Jie Zhang, Tianzhu Guan

**Affiliations:** 1 School of Biology and Food Engineering, Anyang Institute of Technology, Anyang 455000, China; 20160524@ayit.edu.cn; 2 School of Food Science and Engineering, Yangzhou University, Yangzhou 225127, China; mx120241326@stu.yzu.edu.cn (Y.F.); 17870269693@163.com (L.H.); rcx1090161416@163.com (C.R.); wsgtz@163.com (M.G.); 3 College of Food Science and Engineering, Jilin University, Changchun 130062, China

**Keywords:** hederagenin and its glycosides, triterpenoid saponin, anticancer activity, structural modifications, drug discovery

## Abstract

Hederagenin, a pentacyclic triterpenoid saponin from various medicinal plants, shows immense therapeutic potential; however, its inherent low bioavailability severely hinders its clinical translation. This comprehensive review synthesizes recent studies on the health benefits of hederagenin and its glycosides, critically the chemical modification strategies and pharmacological mechanisms aimed at optimizing its bioactivity. Key findings reveal that its broad anticancer and anti-inflammatory activities largely stem from its capacity to modulate crucial cellular signaling pathways, including the NF-κB, PI3K/Akt, and MAPK. Structural modification, particularly intelligent derivatization at the C-28 position, is a central strategy to overcome its pharmacokinetic deficiencies and significantly boost cytotoxicity. Furthermore, its unique pro-oxidant function within cancer cells, achieved by inhibiting the Nrf2-ARE antioxidant pathway, offers a novel approach for selective chemotherapeutics. For the clinical translation of hederagenin, we propose a strategic focus on derivatization through multi-target hybrids and sophisticated delivery systems. This approach is essential for addressing its pharmacokinetic barriers while strategically leveraging its context-dependent pro-oxidant effects.

## 1. Introduction

Natural products, often sourced from “food-medicine homology” practices, are increasingly vital for managing chronic conditions due to their low toxicity and rich content of bioactive compounds like triterpenes and their glycosides [1,2]. Modern research increasingly focuses not only on single active ingredients but also on the synergistic effects of multiple compounds within these traditional resources, as exemplified by the synergistic neuroinflammatory modulation by ginsenosides Rb3 and Rc from *Panax notoginseng* leaves [3]. A prime example is hederagenin, a pentacyclic triterpenoid saponin aglycone first discovered in the seeds of English ivy in 1849 (molecular formula C_30_H_48_O_4_) [4]. Hederagenin has been widely found in many medicinal plants, including the leaves of *Hedera helix* L., the leaves of *Cyclocarya paliurus*, and the fruits of *Fructus Akebiae*, and exists in particularly high levels in the food product quinoa [5,6,7,8]. In recent years, Hederagenin and its glycosides have become a research focus due to their diverse biological activities.

Hederagenin and its glycosides have demonstrated significant pharmacological activities in the treatment of a variety of diseases, including anti-inflammatory, anti-oxidant, anti-tumour, antiviral, antifungal, anti-allergy, anti-haemolysis, anti-parasite, and anti-osteoporosis [9,10]. This broad therapeutic profile is consistent with recent findings for other natural products, where compounds such as polysaccharides have been shown to alleviate intestinal inflammation by modulating intestinal flora [11]. In the realm of anti-tumor research, hederagenin demonstrates substantial inhibitory effects on a multitude of cancer cell lines via mechanisms, including apoptosis induction, inhibition of cell proliferation, modulation of oxidative stress, and regulation of immune responses [12]. While in the field of anti-inflammation, research has demonstrated that hederagenin can exhibit anti-inflammatory properties through the inhibition of macrophage polarization and the subsequent reduction in inflammatory factor release [13]. In addition, hederagenin has shown potential clinical application in regulating the immune system and protecting the cardiovascular and nervous systems [14].

**Figure 1:**
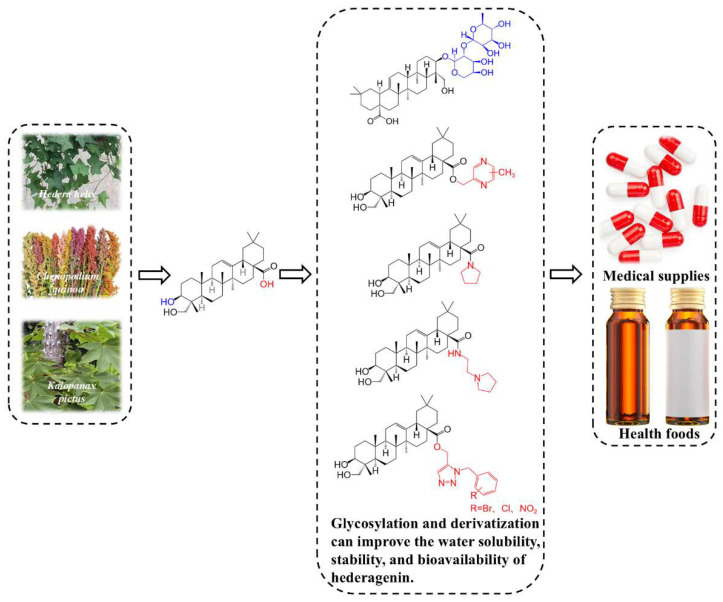
Plant sources, structural modification strategies, and application industries of hederagenin.

Despite diverse pharmacological activities, the clinical application of hederagenin is limited by its poor water solubility, short half-life, and low in vivo bioavailability [15]. This is not an isolated challenge; it represents a common and significant roadblock for a wide array of promising natural small molecules, including polyphenols, alkaloids, and other terpenes, whose clinical potential is often curtailed by these very pharmacokinetic deficiencies [16]. However, chemical modification emerges as an effective strategy to overcome these barriers, optimizing its biological activity and pharmacological effects. The introduction of saccharide moieties, acyl groups, or other functional groups can enhance the water solubility, stability, and bioavailability, consequently augmenting the pharmacological activity [17]. Therefore, in-depth studies on extraction, isolation, and analytical methodologies are essential to expand Hederagenin’s applications in the pharmaceutical and healthcare industries (Figure 1).

Unraveling the complex, multi-target mechanisms of natural compounds like hederagenin increasingly demands sophisticated approaches. Advanced methodologies, such as integrative in silico studies and emerging organ-on-a-chip platforms, are becoming indispensable for deciphering these intricate interactions and providing more accurate predictions of human clinical responses than traditional models [18,19]. In light of these advancements, this review systematically summarizes the extraction methods, structural modification strategies, and established health benefits of hederagenin and its derivatives. This review provides an in-depth discussion of recent progress in its anticancer and anti-inflammatory activities, among other biological effects. Ultimately, this review aims to synthesize current knowledge and provide a forward-looking perspective, offering a scientific framework to guide the future development and clinical application of this promising natural compound.

## 2. Pharmacological Activities and Mechanisms

### 2.1. Chemical Profile of Hederagenin and Its Derivatives

#### 2.1.1. Core Structure and Natural Glycosides

Hederagenin (C_30_H_48_O_4_) is a pentacyclic triterpenoid saponin aglycone that serves as the fundamental scaffold for a diverse family of bioactive compounds. In botanical science, triterpenoids are not only recognized for their pharmacological activities but also serve crucial structural and protective functions. For instance, they are major constituents of the epicuticular wax layer on fruits such as blueberries, where they contribute to physical integrity and defense against environmental stressors [20]. It is naturally abundant in various medicinal plants, most notably English ivy (*Hedera helix*), *Kalopanax pictus*, and quinoa (*Chenopodium quinoa*) [21,22]. Furthermore, recent comprehensive reviews have confirmed its presence in various species of the Jasminum genus, such as *Jasminum officinale*, highlighting its broader distribution across different plant families known for their use in traditional medicine [23].

**Figure 2:**
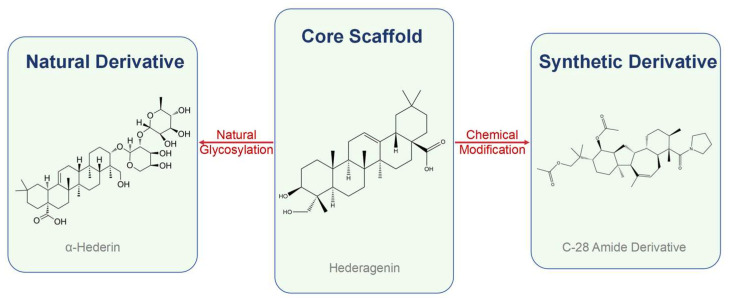
Derivatization strategies from the hederagenin core scaffold. The scaffold can be derivatized through natural glycosylation (e.g., to α-hederin) or through synthetic chemical modifications (e.g., to a C-28 amide derivative) aimed at enhancing pharmacological properties.

In nature, hederagenin frequently exists as glycosides, where sugar moieties are attached to its core structure. This glycosylation is not merely a structural variation but a critical determinant of biological function. Indeed, structure–activity relationship (SAR) studies have provided crucial evidence for the importance of these sugar moieties, showing that the position and type of substitution significantly influence both cytotoxic and hemolytic activities [24]. The chemical structures of the core scaffold and its representative natural and synthetic derivatives are illustrated in Figure 2.

#### 2.1.2. Synthetic Derivatives and Structural Modification

Among the most extensively studied natural glycosides are α-hederin and Macranthoside B (MB), both of which have demonstrated significant pharmacological potential in their own right. However, the natural hederagenin scaffold and its glycosides, while promising, present a classic challenge in drug development: balancing potency with favorable physicochemical properties like bioavailability. Consequently, a major thrust of research has been dedicated to medicinal chemistry, transforming the natural product into a library of optimized drug candidates.

For instance, early efforts focused on modifying the C-28 carboxyl group, with one study creating a series of hederagenin-pyrazine derivatives. This work successfully identified compounds with enhanced cytotoxicity; the most notable derivative exhibited a half-maximal inhibitory concentration (IC_50_) of 3.45 µM against the A549 human lung cancer cell line, a potency comparable to the clinical drug cisplatin [25]. This potency is comparable to that of the first-line clinical drug cisplatin, which typically exhibits an IC_50_ in the 2–10 µM range against A549 cells, highlighting its potential as a therapeutic candidate, though its toxicity against non-malignant cells requires careful consideration. Building on the strategy of C-28 modification, another research group synthesized a series of C-28 amide derivatives and discovered that further acetylating the hydroxyl groups on the A-ring significantly boosted antiproliferative activity.

Their most potent compound, an acetylated amide bearing a pyrrolidinyl group, displayed a remarkable EC_50_ value of 0.4 µM against the A2780 ovarian cancer cell line, making it significantly more potent than both the parent hederagenin and the control drug, betulinic acid [26]. While this value is a significant improvement over the parent aglycone, it still lags behind certain clinical agents for ovarian cancer, such as paclitaxel, which often has IC_50_ values in the nanomolar range. However, the true value of this derivative lies in its remarkable selectivity. Its EC_50_ against non-malignant fibroblasts (NIH3T3) was 9.6 µM, affording a selectivity index (SI) of 24. This ability to dramatically enhance potency while mitigating toxicity to normal cells directly addresses a primary limitation of saponin-based therapeutics and is a critical indicator of its therapeutic potential. This general approach of using hederagenin as a template was further validated by the development of ester derivatives that were up to 30 times more active than hederagenin itself [27].

**Figure 3:**
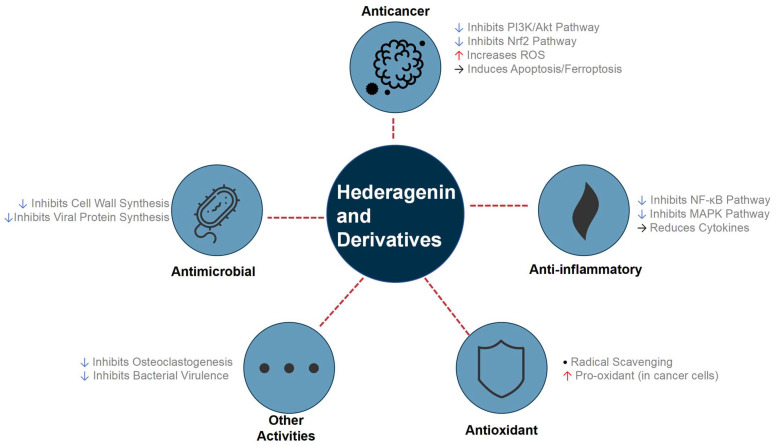
Overview of the main pharmacological activities and pathway regulation of hederagenin and its derivatives. This molecular family demonstrates significant therapeutic potential in multiple areas by up- or down-regulating key signaling pathways.

More sophisticated strategies have involved creating hybrid molecules, such as adding NO-donor moieties to α-hederin to achieve synergistic antiproliferative effects and superior tumor inhibition in vivo [28]. Advanced synthetic methods were also employed, such as the Huisgen 1,3-dipolar cycloaddition, to create novel triazolyl derivatives with potent anticancer activity [29]. Further structural modifications, particularly on the C ring, led to new derivatives with highly selective anti-hepatoma activities [30]. This body of work, with key examples summarized in Table 1, demonstrates a clear and successful trend: targeted chemical derivatization is a powerful and indispensable strategy for optimizing the therapeutic potential of the hederagenin scaffold, which encompasses a broad spectrum of pharmacological activities as detailed in Figure 3.

### 2.2. Anticancer Activity

A predominant theme in the anticancer research of hederagenin is its ability to trigger programmed cell death, with the mitochondrial pathway emerging as a convergent point of action. This pro-apoptotic effect is not driven by a single mechanism but rather by hederagenin’s function as a multi-target agent that modulates various upstream signaling cascades. Initial studies established this mechanism in breast cancer and leukemia cells, where saponins like α-hederin and Macranthoside B (MB) were shown to reduce mitochondrial membrane potential, release cytochrome c, and activate the caspase cascade [31,32]. This fundamental mechanism was subsequently validated in a broader range of malignancies, including lung cancer and colon cancer, where hederagenin itself effectively induced apoptosis by disrupting mitochondrial potential and modulating key regulatory proteins like Bax and Bcl-2 [33,34]. Further investigations into MB confirmed its robust ability to induce mitochondrion-mediated apoptosis in various cancer cells, including ovarian carcinoma [35,36], while research on α-hederin in hepatocellular carcinoma (HCC) further refined our understanding by linking its pro-apoptotic effects to the generation of reactive oxygen species (ROS) [37]. Further investigations revealed that these actions are often initiated higher up in the cell’s signaling pathways. For instance, in glioma cells, hederagenin’s effects were traced to the modulation of the Nur77/PI3K/AKT pathway [38]. The PI3K/Akt signaling pathway was also identified as a key target for MB in various cancer cells [37]. In a distinct but complementary mechanism, α-hederin was found to induce not only apoptosis but also ferroptosis—a different form of programmed cell death—in non-small-cell lung cancer (NSCLC) by disrupting the cellular glutathione system, thereby causing overwhelming oxidative stress [39]. However, a significant caveat underlies these promising mechanistic findings: the encouraging in vitro results are often achieved at micromolar (μM) concentrations, a level that is difficult to attain in vivo, reinforcing the centrality of the bioavailability challenge.

Beyond mechanistic studies, practical considerations such as the botanical source and drug delivery strategies are crucial for translating preclinical findings into viable therapies. Research has shown that the specific source and glycosylation pattern can confer additional benefits, such as the significant antimutagenic activity observed in compounds from *Kalopanax pictus*, suggesting a chemopreventive role [40]. To address the persistent challenge of poor bioavailability, innovative drug delivery systems, such as targeted diblock copolymer-based micelles, have been engineered. While these nanocarriers successfully protected the compound and enhanced its targeted delivery in cell culture models, resulting in superior anticancer effects compared to the free drug, their efficacy in more complex in vivo tumor models, which better replicate physiological barriers and the tumor microenvironment, remains a critical next step for validation [41].

### 2.3. Anti-Inflammatory Activity

Parallel to its anticancer properties, the anti-inflammatory potential of the hederagenin family is equally robust, primarily attributed to its capacity to suppress master inflammatory regulators like NF-κB and MAPKs, which are foundational signaling cascades known to modulate inflammatory responses and drive the pathogenesis of various inflammatory diseases [42,43,44]. Although a foundational study demonstrated potent inhibition of inflammatory mediators in LPS-stimulated macrophages, it is important to note that this in vitro model does not fully replicate the complex cellular and signaling environment of chronic inflammatory diseases like osteoarthritis, necessitating validation in more advanced models [45]. This core mechanism—the suppression of NF-κB and MAPK pathways—was later shown to be effective in more complex disease models, including osteoarthritis, where Hederacoside-C (HDC) protects chondrocytes from degradation [46], and in both bacterial mastitis and acute lung inflammation, where HDC effectively controls the inflammatory response [47,48]. Hederagenin itself has also been validated as a promising anti-osteoarthritis agent, acting through the JAK2/STAT3/MAPK pathway to protect cartilage in vivo [49].

Beyond these general anti-inflammatory actions, the therapeutic potential of these compounds extends to immunomodulation in allergic diseases. In a rat model of allergic asthma, for instance, pretreatment with α-hederin demonstrated preventive effects by modulating key inflammatory and regulatory pathways, including the downregulation of IL-17, suggesting a promising role for hederagenin derivatives in managing specific allergic inflammatory responses [50]. Further research has revealed more nuanced anti-inflammatory mechanisms beyond these canonical pathways. Crucially, some studies have moved beyond observing downstream effects to identify direct molecular targets. A prime example is the saponin SMG-1, which was identified as a direct antagonist of the N-formylmethionyl-leucyl-phenylalanine (FMLP) receptor on neutrophils [22]. This finding is significant as it provides a precise, receptor-level explanation for its ability to control neutrophilic inflammation, distinguishing it from broader, less specific pathway modulations. In animal models of systemic arthritis, which provide a more integrative view of a compound’s effect than simple cell cultures, α-hederin methyl ester showed significant efficacy, supporting its potential for systemic anti-inflammatory applications [51]. In the context of inflammatory bowel disease, Hederacoside-C was found to ameliorate colitis not just by inhibiting cytokines, but also by downregulating S100A9, a key protein in neutrophil degranulation, thereby helping to repair the intestinal barrier [52]. This versatility of action, from receptor-level inhibition to advanced nanomedicine formulations for atopic dermatitis [53], underscores the molecule’s broad therapeutic potential, yet consistently highlights that improving bioavailability, either through structural optimization or delivery systems, is essential for translating these findings into clinical significance.

In summary, the diverse anti-inflammatory activities of hederagenin and its glycosylated derivatives are intricately tied to their specific structural modifications. While hederagenin provides the foundational triterpenoid scaffold, the addition of sugar moieties, as seen in Hederacoside-C (HDC), significantly enhances water solubility and modulates target interactions, improving its efficacy against conditions like osteoarthritis and acute inflammation. Conversely, unique glycosylation patterns, such as in Saponin SMG-1, can confer highly specific mechanisms, exemplified by its direct inhibition of the FMLP receptor on neutrophils. Furthermore, chemical modifications like the methyl esterification in α-hederin methyl ester can optimize lipophilicity and metabolic stability, facilitating systemic anti-inflammatory effects. This highlights that subtle alterations in the core structure, the nature of attached glycosides, or other derivatizations are paramount in fine-tuning pharmacokinetic profiles and dictating precise pharmacological engagement with inflammatory pathways, thereby substantiating the observed therapeutic outcomes across the hederagenin family.

### 2.4. Anti-Oxidant Activity

The role of hederagenin in cellular redox balance is intriguingly complex, exhibiting a dual functionality that is context-dependent. On one hand, numerous studies have established hederagenin and its glycosides as potent, conventional antioxidants. They effectively inhibit lipid peroxidation, with activities comparable to standard antioxidants like BHA, and demonstrate strong free radical scavenging capabilities against DPPH, superoxide anions, and hydrogen peroxide [54,55,56]. Although these conventional antioxidant studies demonstrate their chemical potential, their therapeutic relevance is limited, as these simple in vitro chemical assays do not account for the critical physiological barriers of absorption and metabolism that dictate in vivo efficacy.

However, in a fascinating therapeutic paradox, its anticancer mechanism often involves promoting oxidative stress specifically within tumor cells. This was clearly demonstrated in head and neck cancer, where hederagenin induced apoptosis by increasing mitochondrial ROS and depleting glutathione. This pro-oxidant effect was achieved by inhibiting the Nrf2-ARE antioxidant pathway, which cancer cells often hijack to survive. This ability to selectively function as a protective antioxidant in normal contexts while acting as a damaging pro-oxidant in cancer cells is a hallmark of a sophisticated chemotherapeutic agent and a key area for further exploration [57]. The discovery of this dual role significantly enhances hederagenin’s value for drug development and suggests that future research should not be limited to its broad antioxidant properties, but should instead focus on how to amplify its pro-oxidant effect within tumor cells through structural modification to achieve targeted therapy.

To address whether this pro-oxidant effect extends to hederagenin’s glycosides, recent findings indicate that such activity is indeed observed in glycosylated derivatives [58]. For instance, Aq3639, a hederagenin glycoside isolated from Akebia quinata fruits, has been shown to markedly inhibit the proliferation of MCF-7 breast cancer cells by increasing the generation of reactive oxygen species (ROS) and consequently inducing apoptosis in a time-dependent manner. Interestingly, this inhibitory effect (IC_50_ = 13.10 µmol/l) was approximately seven times greater than that of hederagenin itself (IC_50_ = 93.05 µmol/l). This suggests that not only do glycosides exhibit pro-oxidant effects in tumor cells, but in some cases, the glycosylated form may even possess a more pronounced or potent pro-oxidant activity compared to the aglycone, likely due to improved cellular uptake or altered target interactions influenced by the attached sugar moieties.

### 2.5. Antimicrobial Activity

The therapeutic utility of hederagenin glycosides extends into the realm of infectious diseases, where they display broad-spectrum antimicrobial activity, as summarized in Table 2. Evidence suggests a general mechanism of action, possibly related to membrane disruption, which is common for saponins. This is supported by findings of potent antifungal activity against a range of pathogenic fungi, including Candida albicans, where α-hederin’s mode of action was suggested to be similar to the cell-wall-inhibiting drug caspofungin [59,60]. However, a critical comparison is essential when evaluating its clinical potential. While its mechanism of action is novel, its in vitro potency (MICs = 6.25–25 µg/mL) remains modest compared to a first-line clinical antifungal like caspofungin, whose MIC against *C. albicans* is typically below 0.1 µg/mL [61]. This suggests that substantial structural optimization would be necessary to elevate its activity to a clinically relevant level for it to be considered a viable antifungal agent. In addition to antifungal effects, significant antiviral activity has been demonstrated against Enterovirus 71 (EV71) [62]. The antimicrobial profile is completed by studies showing significant antibacterial activity against both Gram-positive and Gram-negative bacteria, with some saponins even surpassing the efficacy of the standard antibiotic gentamycin against *E. faecalis* [63].

A particularly innovative therapeutic strategy involves targeting bacterial virulence rather than viability, and hederagenin has emerged as a promising candidate in this area. It was found to significantly inhibit pneumolysin (PLY), a key pore-forming toxin of Streptococcus pneumoniae. By neutralizing virulence factors, hederagenin can prevent cell lysis and inflammation central to pneumococcal infections. This represents a promising antibiotic-alternative strategy that could reduce the pressure for resistance development [64]. Although these in vitro antimicrobial data show great promise, especially against resistant strains, the hemolytic activity and poor oral absorption common to saponins represent significant hurdles. This suggests that for developing systemic anti-infective agents, research must focus on chemical modifications to reduce toxicity and improve pharmacokinetics, while topical applications may offer a more immediate path forward.

### 2.6. Other Notable Biological Activities

#### 2.6.1. Antiparasitic Activity

The hederagenin scaffold has proven to be a versatile template for developing new anthelmintic agents. A screening of synthetic hederagenin derivatives against the liver fluke Fasciola hepatica identified several active molecules, with one compound showing particular potency and selectivity. This highlights the potential of modified hederagenins as promising candidates for next-generation flukicides to combat parasitic infections [65].

#### 2.6.2. Antiosteoporosis Activity

Finally, hederagenin has shown significant promise in treating osteoclast-mediated bone diseases. By inhibiting RANKL-induced osteoclastogenesis and bone resorption in vitro via the ERK and p38 pathways, and protecting against bone loss in in vivo models, hederagenin demonstrates clear potential for application in the prevention and treatment of osteoporosis [66].

## 3. Discussion

Hederagenin and its glycosides should be viewed not merely as active components in traditional remedies, but as a privileged scaffold whose value is rooted in its broad pharmacological profile and high degree of chemical tractability. However, for greater scientific clarity, it is essential to distinguish between studies demonstrating direct target engagement and those that merely report the modulation of downstream signaling pathways. Much of the current research, particularly in the anticancer and anti-inflammatory fields, falls into the latter category, showing that hederagenin can inhibit cascades like the NF-κB, PI3K/Akt, and MAPK pathways. While valuable, these findings describe the consequences of the drug’s action rather than its primary molecular trigger. In contrast, a smaller but highly significant subset of studies has identified direct molecular targets, such as the inhibition of the FMLP receptor by saponin SMG-1 and the neutralization of the bacterial toxin pneumolysin (PLY). These instances of proven, direct binding provide a much clearer and more robust mechanistic foundation. Therefore, a key future direction for the field should be to shift focus from cataloging downstream effects to identifying the primary protein targets through which hederagenin and its derivatives initiate their diverse pharmacological activities.

Despite compelling preclinical data, the translation of hederagenin into clinical applications is hindered by a primary obstacle: its exceedingly low in vivo bioavailability. To move forward, it is crucial to dissect the specific mechanisms underlying this limitation, which are multi-faceted and extend beyond simple insolubility. The challenge involves at least three key pharmacokinetic barriers: poor absorption, rapid metabolism, and active cellular efflux. Firstly, its poor water solubility fundamentally limits its dissolution in the gastrointestinal tract, representing the initial barrier to oral absorption. Secondly, the compound’s short reported half-life is indicative of rapid metabolic clearance, likely through extensive hepatic metabolism, which prevents sustained therapeutic concentrations from being achieved in the bloodstream. However, perhaps the most critical and sophisticated barrier is its interaction with efflux transporters. Recent evidence strongly suggests that hederagenin is a substrate for P-glycoprotein (P-gp), an efflux pump that actively expels xenobiotics from cells. This means that even if hederagenin is absorbed and reaches target tissues, it is efficiently ejected from the intracellular environment, preventing it from engaging its therapeutic targets. This active efflux mechanism poses a more profound pharmacological challenge than poor solubility alone, as it directly nullifies the drug’s efficacy at the cellular level. Therefore, designing derivatives that can evade or inhibit this P-gp-mediated resistance is not just beneficial but likely essential for unlocking hederagenin’s therapeutic potential.

To address these pharmacokinetic challenges, structural modification has emerged as the principal strategy for enhancing the therapeutic potential of the hederagenin scaffold. Medicinal chemistry efforts have demonstrated that derivatization at the C-28 position, through either amidation or esterification, can dramatically increase cytotoxicity. For instance, the development of certain C-28 amide derivatives led to a more than 25-fold enhancement in cytotoxic potency, while other ester derivatives proved to be up to 30 times more active than the parent hederagenin molecule. However, a critical assessment of the therapeutic prospects of these derivatives requires balancing enhanced potency against toxicity and selectivity. For example, the promise of certain highly active derivatives lies not just in their sub-micromolar EC_50_ values, but more importantly, in their high selectivity index (SI > 24) between cancerous and non-malignant cells. This ability to improve safety while increasing potency is a crucial determinant for the clinical translation of saponin-based drugs, as it directly defines the therapeutic window. The introduction of nitrogen-containing heterocycles, such as pyrazines and triazoles, represents a rational design approach to improve target affinity by introducing new hydrogen bond donors and acceptors. More advanced strategies, however, signal a conceptual shift from optimizing physicochemical properties to designing multi-target therapeutics. The creation of hybrid molecules that couple hederagenin with nitric oxide (NO) donor moieties exemplifies this, aiming for synergistic effects where the scaffold provides the cytotoxic punch and the NO group modulates the tumor microenvironment. This move from simple derivatization to intelligent, multi-target drug design represents a sophisticated evolution in the compound’s development.

Beyond the critical issue of bioavailability, a frank assessment of hederagenin’s path to clinical use must also consider significant practical and logistical limitations that are often overlooked in preclinical studies. The synthesis of the most potent derivatives, while successful at the laboratory scale, frequently involves multi-step reaction sequences that may not be readily amenable to large-scale, cost-effective industrial production. Challenges in achieving high yields, the need for expensive reagents or catalysts, and complex purification processes can render the final active pharmaceutical ingredient (API) prohibitively expensive. Furthermore, the reliance on hederagenin as a starting scaffold is itself a potential bottleneck. While sourced from several plants, variations in crop yield, geographical location, and extraction efficiency can lead to inconsistencies in the supply and quality of the raw material. These real-world constraints of synthetic scalability and source material availability represent a formidable, non-biological hurdle that must be addressed through the development of more efficient synthetic routes or alternative production methods, such as metabolic engineering in microbial hosts, to ensure the viable translation of these promising compounds from the bench to the bedside.

Looking forward, several knowledge gaps must be addressed to fully unlock hederagenin’s potential. A particularly intriguing area is its dual antioxidant/pro-oxidant activity. While protective in normal cells, hederagenin functions as a pro-oxidant in cancer cells, inducing apoptosis by increasing reactive oxygen species and inhibiting the Nrf2 antioxidant defense pathway that cancer cells rely on for survival. Future research should focus not on its conventional antioxidant properties, but on strategies to selectively amplify this pro-oxidant mechanism within the tumor microenvironment for a more targeted chemotherapeutic effect. Furthermore, a highly promising and timely avenue for future research is the interaction between hederagenin, its glycosides, and the gut microbiome. Given that saponins generally exhibit poor oral absorption, they persist in the gastrointestinal tract, creating a prolonged opportunity for direct interaction with gut microbiota. It is plausible that some of the observed systemic anti-inflammatory and metabolic benefits of these compounds are not solely due to the small fraction that is absorbed, but are also mediated indirectly through the modulation of the gut microbiome, a mechanism of action that has been increasingly reported for other structurally related triterpenoid saponins, such as ginsenosides, in the context of both cancer and inflammatory bowel disease [67,68].

Hederagenin could potentially alter the microbial composition, promoting the growth of beneficial bacteria or inhibiting pro-inflammatory species, thereby improving intestinal barrier integrity and reducing systemic inflammation linked to conditions like inflammatory bowel disease and metabolic syndrome. Investigating this gut-centric mechanism of action could reframe our understanding of hederagenin’s therapeutic effects and open up new applications for oral formulations. Furthermore, given hederagenin’s ability to modulate key signaling pathways like Wnt/β-catenin, its potential application in new fields such as treating androgenetic alopecia is also worth exploring, as these pathways are core targets for other natural products in treating this condition [69,70]. Concurrently, formulation science must advance beyond simple encapsulation. Advanced delivery systems, such as nanoemulsions, are increasingly recognized as a powerful strategy to enhance the stability and bioavailability of lipophilic bioactive compounds [71]. Notably, natural saponins themselves are effective emulsifiers, suggesting a particularly promising strategy wherein hederagenin or its derivatives could be formulated into stable nanoemulsions to overcome solubility and absorption challenges. While targeted micelles are a promising start, the next generation of drug delivery systems should incorporate active targeting ligands for receptors overexpressed on cancer cells and be evaluated for their ability to alter the compound’s metabolic fate. Finally, bridging the persistent gap between in vitro results and clinical reality requires the adoption of cutting-edge methodologies. The use of integrative in silico studies can help systematically unravel the compound’s multi-target interactions, while organ-on-a-chip platforms offer a superior model for predicting human pharmacokinetics and toxicology, thereby de-risking and accelerating the journey toward clinical translation.

## 4. Materials and Methods

This review is based on a comprehensive literature search conducted across major scientific databases, including PubMed, Scopus, Web of Science, ScienceDirect, SpringerLink, Wiley Online Library, and MDPI. Search queries were constructed using the primary terms “Hederagenin,” “α-hederin,” “hederagenin glycosides,” and “Macranthoside B”. These keywords were used in various Boolean combinations with secondary terms related to its botanical sources (e.g., *Hedera helix*, *Kalopanax pictus*, and *Chenopodium quinoa*) and pharmacological properties (e.g., “anticancer,” “anti-inflammatory,” “antioxidant,” “cytotoxicity,” “synthesis,” “derivatives,” and “pharmacological mechanism”). No date limitations were applied to ensure the inclusion of both foundational and recent studies.

To ensure the relevance and quality of the included literature, the following inclusion criteria were established: (1) original research articles, reviews, and reports focusing on hederagenin and its natural or synthetic glycosides; (2) studies investigating its pharmacological activities, structural modifications, analytical methods, or mechanisms of action; (3) articles published in the English language. Exclusion criteria included articles where hederagenin was a minor component of a complex extract without specific investigation, non-peer-reviewed sources, and conference abstracts lacking sufficient data.

The study selection was performed in a two-stage process. The process was conducted independently by two authors (Guangjie Zhang and Yining Feng) to ensure objectivity. First, titles and abstracts of the retrieved articles were screened for relevance against the inclusion criteria. Second, the full texts of potentially eligible articles were thoroughly reviewed to make the final selection. Additionally, the reference lists of all included articles were manually screened to identify any further relevant publications. This approach ensured that a comprehensive and relevant body of literature was selected for this review.

## 5. Conclusions

Hederagenin has been firmly established as a highly valuable natural scaffold, possessing a remarkable diversity of potent pharmacological activities. These include significant anticancer, anti-inflammatory, antioxidant, and antimicrobial effects, underscoring its broad therapeutic potential against a wide spectrum of pathological conditions. Particularly noteworthy is its sophisticated dual functionality, acting as a protective antioxidant in normal physiological contexts while functioning as a targeted pro-oxidant within cancer cells by inhibiting the Nrf2 antioxidant defense pathway.

Despite this promise, its translation into a clinical therapeutic is fundamentally hindered by critical pharmacokinetic barriers, including poor water solubility, a short metabolic half-life, and active efflux from target cells. Therefore, the successful transition of hederagenin from a promising preclinical compound to an effective clinical therapy hinges not on the continued discovery of new biological effects, but on a strategic focus on intelligent structural modification and advanced drug delivery systems engineered to conquer these well-defined pharmacokinetic barriers. Successfully overcoming these challenges would unlock the full potential of hederagenin, paving the way for its development as a powerful, multi-target agent for treating complex diseases ranging from cancer to chronic inflammatory conditions.

## Figures and Tables

**Table 1:** Summary of key structural modification strategies for hederagenin and the pharmacological outcomes of their derivatives.

Modification Strategy	Representative Compound(s)	Key Findings and Quantitative Metrics	Bioavailability/ Toxicity Notes	Key Tested Models	Reference
Hederagenin-pyrazine derivatives	Compound **9** (2,6-dimethylpyrazine derivative)	Most derivatives were more potent than the parent compound. Compound **9** showed cytotoxicity comparable to cisplatin against A549 cells (IC_50_ = 3.45 µM) while exhibiting lower toxicity to normal cardiomyocytes (H9c2 IC_50_ = 16.69 µM).	Compound **9** demonstrated favorable selectivity, being less toxic to normal heart (H9c2) and kidney (MDCK) cells compared to the standard drug cisplatin.	A549 (NSCLC), HepG2 (hepatocellular carcinoma), MCF-7 (breast)	[25]
C-28 amide derivatives	Compound **2c** (Acetylated-pyrrolidinyl amide)	Acetylation of C-3/C-23 hydroxyls generally enhanced cytotoxicity (>25-fold). Compound **2c** was the most potent, with high selectivity against A2780 cells (EC_50_ = 0.4 µM) vs. non-malignant fibroblasts (NIH3T3 EC_50_ = 9.6 µM; SI = 24).	Acetylation of the A-ring was a key strategy to increase potency and selectivity. The hydroxylated precursor (**1c**) was potent but also toxic to normal cells, whereas the acetylated derivative (**2c**) showed significantly improved selectivity.	A2780 (ovarian), FaDu (hypopharyngeal), HT29 (colon), SW1736 (thyroid)	[26]
Ester derivatives	Compound **28** (Ethylpyrrolidinyl amide	Amide derivatives were generally more potent than esters. Compound **28** was among the most active (approx. 30-fold > He), with an EC_50_ of 1.1 µM against A2780 cells.	No direct toxicity data against normal cells was provided, but the study highlights that C-28 amidation, particularly with N-heterocycles, is a highly effective strategy for boosting potency.	Panel of six including A2780 (ovarian), 518A2 (melanoma), and HT29 (colon)	[27]
Hybrid molecules with NO-donor moieties	Compound **5c** (PSD-C_4_; linker-NO donor)	PSD derivatives were more potent than α-hederin derivatives. Compound **5c** showed strong in vitro activity (IC_50_ = 5.4–10.7 µM) and superior in vivo tumor inhibition compared to Taxol (27.4% vs. 23.3%).	To overcome poor solubility for in vivo studies, compound **5c** was formulated into polymer micelles. The formulation showed no deaths in mice, suggesting lower toxicity compared to the parent saponin (PSD).	In vitro: HCT-116 (colon), NCI-H460 (lung), SMMC-7721 (liver)	[28]
Triazolyl derivatives	Compound **11** (ortho-fluorobenzyl-triazolyl ester)	Ester derivatives were generally more potent than amides. Compound **11** was the most cytotoxic against HT29 cells (EC_50_ = 1.6 µM) and showed good selectivity vs. normal fibroblasts (NIH 3T3 EC_50_ = 8.7 µM, SI = 5.4).	The introduction of a triazole ring via click chemistry is an effective method for generating libraries of potent compounds. Ester derivatives were generally more cytotoxic than the corresponding amides.	HT29 (colon), A2780 (ovarian), A549 (lung), and others	[29]
C ring-modified derivatives	Compound **5** (12-hydroxyimino derivative)	C-ring modification significantly enhanced activity. Compound **5** was the most potent (IC_50_ = 1.88 µM), surpassing 5-fluorouracil, and showed high selectivity over normal L929 cells (IC_50_ = 21.23 µM, SI ≈ 11.3).	This strategy aimed to circumvent the high hemolytic toxicity of natural saponins by modifying the aglycone. All derivatives showed low toxicity to normal cells.	HepG2 (hepatocellular carcinoma), L929 (normal fibroblast)	[30]

**Table 2:** Summary of the antimicrobial activities of hederagenin and its derivatives.

Compound Category	Activity Type	Key Pathogens	Key Findings and Quantitative Metrics (MIC/EC_50_)	Proposed Mechanism of Action	References
Monodesmosides (α-hederin, sapindoside B) from *Kalopanax pictum*	Antifungal	*C. albicans*, *M. canis*, *T. mentagrophytes*, and other pathogenic fungi.	Exhibited significant antifungal activity (MICs = 6.25–25 µg/mL), whereas the parent bisdesmosides were inactive. The presence of a free C-28 carboxyl group is essential for activity.	A haploinsufficiency screen of α-hederin against S. cerevisiae revealed a profile similar to the drug caspofungin, suggesting inhibition of fungal cell wall (β(1,3)-D-glucan) synthesis.	[59,60]
Hederasaponin B from *Hedera helix*	Antiviral	Enterovirus 71 (EV71), specifically subgenotypes C3 and C4a.	Showed significant antiviral activity against both EV71-C3 (EC_50_ = 24.77 µg/mL) and EV71-C4a (EC_50_ = 41.77 µg/mL) by reducing the formation of cytopathic effects (CPEs).	Inhibited the expression of the viral capsid protein VP2 at the post-entry stage, suggesting a targeted inhibition of viral protein synthesis or assembly.	[62]
Hederagenin glycosides from *Cephalaria elmaliensis*	Antibacterial	A broad spectrum including Gram-positive (*S. aureus*, *E. faecalis*) and Gram-negative (*E. coli*, *P. aeruginosa*) bacteria.	All tested saponins were highly active. They showed exceptionally strong activity against *E. faecalis* (MICs = 1–8 µg/mL), surpassing the standard antibiotic gentamicin (MIC = 16.0 µg/mL).	Mechanism not specified, but likely involves membrane disruption. As amphiphilic molecules, saponins can intercalate into the bacterial cell membrane, leading to pore formation, increased permeability, and eventual cell lysis.	[63]

## Data Availability

Not applicable.

## Abbreviations

The following abbreviations are used in this manuscript:

EV71	Enterovirus 71
HCC	Hepatocellular carcinoma
HDC	Hederacoside-C
MAPKs	Mitogen-activated protein kinase
MB	Macranthoside B
NSCLC	Non-small-cell lung cancer
PLY	Pneumolysin
ROS	Reactive oxygen species

## References

[1] Sun-Waterhouse D.-X., Chen X.-Y., Liu Z.-H., Waterhouse G.I.N., Kang W.-Y. (2024). Transformation from traditional medicine-food homology to modern food-medicine homology. Food Med. Homol..

[2] Hao X.-T., Peng R., Guan M., Zhang H.-J., Guo Y., Shalapy N.M., Liu X.-Q., Ma C.-Y. (2024). The food and medicinal homological resources benefiting patients with hyperlipidemia: Categories, functional components, and mechanisms. Food Med. Homol..

[3] Ran S.-Z., Peng R., Guo Q.-W., Ma L.-J., Wan J.-B., Huang J.-Q., Wang Z.-Y., Chen G. (2025). Synergistic Effects of Ginsenoside Rb3 and Rc From Panax notoginseng Leaf on Alleviating Neuroinflammation via PACAP in Depression. Food Front..

[4] Geng Z., Wang Y., Ma M., Wei Y., Xie W., Cheng J., Chen Y., Fang X., Wang H., Bi Y. (2025). Discovery and biological evaluation of hederagenin derivatives as non-substrate inhibitors of P-glycoprotein-mediated multidrug resistance. Eur. J. Med. Chem..

[5] Zhou M., Quek S.Y., Shang X., Fang S. (2021). Geographical variations of triterpenoid contents in Cyclocarya paliurus leaves and their inhibitory effects on HeLa cells. Ind. Crops Prod..

[6] Lin R., Liu L., Silva M., Fang J., Zhou Z., Wang H., Xu J., Li T., Zheng W. (2021). Hederagenin Protects PC12 Cells Against Corticosterone-Induced Injury by the Activation of the PI3K/AKT Pathway. Front. Pharmacol..

[7] Carpio-Paucar G.N., Palo-Cardenas A.I., Rondón-Ortiz A.N., Pino-Figueroa A., Gonzales-Condori E.G., Villanueva-Salas J.A. (2023). Cytotoxic Activity of Saponins and Sapogenins Isolated from *Chenopodium quinoa* Willd. in Cancer Cell Lines. Scientifica.

[8] Li Y., Yang Z., Jin C., Liu Y., Zhang Y., Ma X., Song W., Cai R., Feng K., Zhao W. (2024). Akebia fruit: A review on its extraction, phytochemicals, bioactivities and applications. eFood.

[9] Yu T., Cheng H., Li X., Huang W., Li H., Gao X., Zhao J., Zhang X., Gu X., Bi Y. (2023). Design and synthesis of hederagenin derivatives modulating STING/NF-κBsignaling for the relief of acute liver injury in septic mice. Eur. J. Med. Chem..

[10] Yang M., Wang J., Wang Q. (2022). Hederagenin Exerts Potential Antilipemic Effect via p38MAPK Pathway in Oleic Acid-induced HepG2 cells and in Hyperlipidemic Rats. An. Da Acad. Bras. De Ciências.

[11] Ning E.-J., Sun C.-W., Wang X.-F., Chen L., Li F.-F., Zhang L.-X., Wang L.-P., Ma Y.-N., Zhu J., Li X. (2024). Artemisia argyi polysaccharide alleviates intestinal inflammation and intestinal flora dysbiosis in lipopolysaccharide-treated mice. Food Med. Homol..

[12] Xie W., Fang X., Li H., Lu X., Yang D., Han S., Bi Y. (2023). Advances in the anti-tumor potential of hederagenin and its analogs. Eur. J. Pharmacol..

[13] Wang L., Zhao M. (2022). Suppression of NOD-like receptor protein 3 inflammasome activation and macrophage M1 polarization by hederagenin contributes to attenuation of sepsis-induced acute lung injury in rats. Bioengineered.

[14] Yang X., Li G., Chen L., Zhang C., Wan X., Xu J. (2011). Quantitative determination of hederagenin in rat plasma and cerebrospinal fluid by ultra fast liquid chromatography–tandem mass spectrometry method. J. Chromatogr. B.

[15] Zeng J., Huang T., Xue M., Chen J., Feng L., Du R., Feng Y. (2018). Current knowledge and development of hederagenin as a promising medicinal agent: A comprehensive review. RSC Adv..

[16] Ye F., Zheng S., Shan S., Cai J., Liu Y., Chen W., He X., Zhao C. (2025). Delivery of Natural Small Molecules Through Nanocarriers for Cancer Treatment. Food Front..

[17] Zhang H., Li Y., Liu Y. (2024). An updated review of the pharmacological effects and potential mechanisms of hederagenin and its derivatives. Front. Pharmacol..

[18] Ren C.-X., Gao M.-Y., Li N., Tang C., Chu G.-H., Yusuf A., Xiao L.-X., Yang Z.-Q., Guan T.-Z. (2024). Identification and mechanism elucidation of medicative diet for food therapy XQCSY in NAFLD prevention: An integrative in silico study. Food Med. Homol..

[19] Wang X., Miao Y.-H., Zhao X.-M., Liu X., Hu Y.-W., Deng D.-W. (2024). Perspectives on organ-on-a-chip technology for natural products evaluation. Food Med. Homol..

[20] Ding M., Zong Z., Han Y., Wang L., Kong Q., Chen H., Xiao S., Liu R., Wu W., Gao H. (2024). Impact of lipase treatment on cuticle wax structure and anthocyanin metabolism in postharvest blueberries. Food Front..

[21] Jayasinghe L., Shimada H., Hara N., Fujimoto Y. (1995). Hederagenin glycosides from *Pometia eximia*. Phytochemistry.

[22] Hwang T.L., Wang C.C., Kuo Y.H., Huang H.C., Wu Y.C., Kuo L.M., Wu Y.H. (2010). The hederagenin saponin SMG-1 is a natural FMLP receptor inhibitor that suppresses human neutrophil activation. Biochem. Pharmacol..

[23] Rescigno A., Zucca P., Peddio S., Srikanth S., Kaushik N.P., Kumar N.V.A., Leyva-Gómez G., Kregiel D., Abu-Reidah I.M., Sen S. (2025). Harnessing Jasminum Bioactive Compounds: Updated Insights for Therapeutic and Food Preservation Innovations. Food Front..

[24] Chwalek M., Lalun N., Bobichon H., Plé K., Voutquenne-Nazabadioko L. (2006). Structure–activity relationships of some hederagenin diglycosides: Haemolysis, cytotoxicity and apoptosis induction. Biochim. Biophys. Acta (BBA)-Gen. Subj..

[25] Fang K., Zhang X.-H., Han Y.-T., Wu G.-R., Cai D.-S., Xue N.-N., Guo W.-B., Yang Y.-Q., Chen M., Zhang X.-Y. (2018). Design synthesis and cytotoxic analysis of novel hederagenin–pyrazine derivatives based on partial least squares discriminant analysis. Int. J. Mol. Sci..

[26] Rodríguez-Hernández D., Barbosa L.C., Demuner A.J., Martins J.P.A., Fischer L., Csuk R. (2019). Hederagenin amide derivatives as potential antiproliferative agents. Eur. J. Med. Chem..

[27] Rodríguez-Hernández D., Demuner A.J., Barbosa L.C., Csuk R., Heller L. (2015). Hederagenin as a triterpene template for the development of new antitumor compounds. Eur. J. Med. Chem..

[28] Yang Z., Li H., Li Z., Feng Y., Jin Y., Liu Y., Li M., Liu R., Fang Y. (2020). Improved antiproliferative activity of novel Pulsatilla saponin D/α-hederin derivatives containing nitric oxide donors. New J. Chem..

[29] Rodríguez-Hernández D., Demuner A.J., Barbosa L.C., Heller L., Csuk R. (2016). Novel hederagenin–triazolyl derivatives as potential anti-cancer agents. Eur. J. Med. Chem..

[30] Liu X., Sun L., Liu Q.-H., Chen B.-Q., Liu Y.-M. (2020). Synthesis, characterization and anti-hepatoma activity of new hederagenin derivatives. Mini Rev. Med. Chem..

[31] Cheng L., Shi L., Wu J., Zhou X., Li X., Sun X., Zhu L., Xia T.S., Ding Q. (2018). A hederagenin saponin isolated from Clematis ganpiniana induces apoptosis in breast cancer cells via the mitochondrial pathway. Oncol. Lett..

[32] Cheng L., Xia T.-S., Wang Y.-F., Zhou W., Liang X.-Q., Xue J.-Q., Shi L., Wang Y., Ding Q., Wang M. (2014). The anticancer effect and mechanism of α-hederin on breast cancer cells. Int. J. Oncol..

[33] Gao Y., He C., Bi W., Wu G., Altman E. (2016). Bioassay guided fractionation identified hederagenin as a major cytotoxic agent from Cyclocarya paliurus leaves. Planta Medica.

[34] Liu B.-X.-Z., Zhou J.-Y., Li Y., Zou X., Wu J., Gu J.-F., Yuan J.-R., Zhao B.-J., Feng L., Jia X.-B. (2014). Hederagenin from the leaves of ivy (*Hedera helix* L.) induces apoptosis in human LoVo colon cells through the mitochondrial pathway. BMC Complement. Altern. Med..

[35] Shan Y., Guan F., Zhao X., Wang M., Chen Y., Wang Q., Feng X. (2016). Macranthoside B induces apoptosis and autophagy via reactive oxygen species accumulation in human ovarian cancer A2780 cells. Nutr. Cancer.

[36] Wang J., Zhao X.-Z., Qi Q., Tao L., Zhao Q., Mu R., Gu H.-Y., Wang M., Feng X., Guo Q.-L. (2009). Macranthoside B, a hederagenin saponin extracted from Lonicera macranthoides and its anti-tumor activities in vitro and in vivo. Food Chem. Toxicol..

[37] Li Y., Li M., Ahmed K., Yang J., Song L., Cui Z., Hiraku Y. (2022). Mechanistic Study of Macranthoside B Effects on Apoptotic Cell Death in Human Cervical Adenocarcinoma Cells. Folia Biol..

[38] Dai Y., Masra N., Zhou L., Yu C., Jin W., Ni H. (2023). Hederagenin suppresses glioma cell biological activities via Nur77 in vitro study. Food Sci. Nutr..

[39] Wu Y., Wang D., Lou Y., Liu X., Huang P., Jin M., Huang G. (2022). Regulatory mechanism of α-hederin upon cisplatin sensibility in NSCLC at safe dose by destroying GSS/GSH/GPX2 axis–mediated glutathione oxidation-reduction system. Biomed. Pharmacother..

[40] Lee K.-T., Sohn I.-C., Park H.-J., Kim D.-W., Jung G.-O., Park K.-Y. (2000). Essential moiety for antimutagenic and cytotoxic activity of hederagenin monodesmosides and bisdesmosides isolated from the stem bark of *Kalopanax pictus*. Planta Medica.

[41] Sun J., Liu T., Xu J. (2016). Improving the anticancer activity of α-hederin by physically encapsulating it with targeted micelles assembled from amphiphilic block copolymers. J. Drug Deliv. Sci. Technol..

[42] Liu Z., Yao X., Jiang W., Li W., Zhu S., Liao C., Zou L., Ding R., Chen J. (2020). Advanced oxidation protein products induce microglia-mediated neuroinflammation via MAPKs-NF-κB signaling pathway and pyroptosis after secondary spinal cord injury. J. Neuroinflamm..

[43] Yang S., Li F., Lu S., Ren L., Bian S., Liu M., Zhao D., Wang S., Wang J. (2022). Ginseng root extract attenuates inflammation by inhibiting the MAPK/NF-κB signaling pathway and activating autophagy and p62-Nrf2-Keap1 signaling in vitro and in vivo. J Ethnopharmacol..

[44] Cai Y.-K., Sun J.-Y., Chen Y.-Y., Zhang M.-Q., Sun S.-T., Ren Q.-D., Wang M.-X., Farag M.A., Zhang B., Guo X. (2025). Millet Bran Bound Phenolic Compounds Suppresses LPS-Induced Inflammatory Response in Macrophages and Liver Injury Mice via TLR4/NF-κB Signaling Pathway. eFood.

[45] Kim Y.-K., Kim R.-G., Park S.-J., Ha J.-H., Choi J.-W., Park H.-J., Lee K.-T. (2002). In vitro antiinflammatory activity of kalopanaxsaponin A isolated from *Kalopanax pictus* in murine macrophage RAW 264.7 cells. Biol. Pharm. Bull..

[46] Xu H.-C., Wu B., Ma Y.-M., Xu H., Shen Z.-H., Chen S. (2020). Hederacoside-C protects against AGEs-induced ECM degradation in mice chondrocytes. Int. Immunopharmacol..

[47] Akhtar M., Shaukat A., Zahoor A., Chen Y., Wang Y., Yang M., Umar T., Guo M., Deng G. (2020). Hederacoside-C inhibition of Staphylococcus aureus-induced mastitis via TLR2 & TLR4 and their downstream signaling NF-κB and MAPKs pathways in vivo and in vitro. Inflammation.

[48] Akhtar M., Shaukat A., Zahoor A., Chen Y., Wang Y., Yang M., Umar T., Guo M., Deng G. (2019). Anti-inflammatory effects of Hederacoside-C on Staphylococcus aureus induced inflammation via TLRs and their downstream signal pathway in vivo and in vitro. Microb. Pathog..

[49] Shen Y., Teng L., Qu Y., Huang Y., Peng Y., Tang M., Fu Q. (2023). Hederagenin suppresses inflammation and cartilage degradation to ameliorate the progression of osteoarthritis: An in vivo and in vitro study. Inflammation.

[50] Ebrahimi H., Fallahi M., Khamaneh A.M., Saadatlou M.A.E., Saadat S., Keyhanmanesh R. (2016). Effect of a-Hederin on IL-2 and IL-17 mRNA and miRNA-133a Levels in Lungs of Ovalbumin-Sensitized Male Rats. Drug Dev. Res..

[51] Da W.L., Hyun J.E., Jeong C.S., Kim Y.S., Lee E.B. (2003). Antiinflammatory activity of α-hederin methyl ester from the alkaline hydrolysate of the butanol fraction of *Kalopanax pictus* bark extract. Biol. Pharm. Bull..

[52] Zha Z.-X., Lin Y., Wang K.-X., Zhang Y.-L., Li D., Xu G.-Q., Xu Q.-M., Liu Y.-L. (2023). Hederacoside C ameliorates colitis via restoring impaired intestinal barrier through moderating S100A9/MAPK and neutrophil recruitment inactivation. Acta Pharmacol. Sin..

[53] Lee K.-J., Ratih K., Kim G.-J., Lee Y.-R., Shin J.-S., Chung K.-H., Choi E.-J., Kim E.-K., An J.H. (2022). Immunomodulatory and anti-inflammatory efficacy of hederagenin-coated maghemite (γ-Fe2O3) nanoparticles in an atopic dermatitis model. Colloids Surf. B Biointerfaces.

[54] Gülçin İ., Mshvildadze V., Gepdiremen A., Elias R. (2004). Antioxidant activity of saponins isolated from ivy: α-hederin hederasaponin-C, hederacolchiside-E and hederacolchiside-F. Planta Medica.

[55] Gülçin İ., Mshvildadze V., Gepdiremen A., Elias R. (2006). The antioxidant activity of a triterpenoid glycoside isolated from the berries of Hedera colchica: 3-O-(β-d-glucopyranosyl)-hederagenin. Phytother. Res. Int. J. Devoted Pharmacol. Toxicol. Eval. Nat. Prod. Deriv..

[56] Oladimeji A.O., Oladosu I.A., Ali M.S., Khan S.A., Yousuf S. (2016). Cytotoxic effect of hederagenin on NCI-H460, human non-small lung cancer cells and its free radical scavenging activities. J. Biol. Act. Prod. Nat..

[57] Kim E.H., Baek S., Shin D., Lee J., Roh J.-L. (2017). Hederagenin Induces Apoptosis in Cisplatin-Resistant Head and Neck Cancer Cells by Inhibiting the Nrf2-ARE Antioxidant Pathway. Oxidative Med. Cell. Longev..

[58] Sato A., Masaka S., Aisaka A., Ishibashi I., Yabuki A., Nemoto H., Ohira M., Yamaura M., Suzuki K., Matsuzaki K. (2025). Hederagenin Glycoside Isolated from the Pericarps of Akebia quinata Fruits Induces Apoptotic Cell Death in Breast Cancer Cells. Anticancer Res..

[59] Lee M.-W., Kim S.U., Hahn D.-R. (2001). Antifungal activity of modified hederagenin glycosides from the leaves of Kalopanax pictum var chinense. Biol. Pharm. Bull..

[60] Prescott T.A., Rigby L.P., Veitch N.C., Simmonds M.S. (2014). The haploinsufficiency profile of α-hederin suggests a caspofungin-like antifungal mode of action. Phytochemistry.

[61] Li F., Zhou M., Jiao Z., Zou Z., Yu E., He Z. (2021). Caspofungin pharmacokinetics and probability of target attainment in ICU patients in China. J. Glob. Antimicrob. Resist..

[62] Song J., Yeo S.-G., Hong E.-H., Lee B.-R., Kim J.-W., Kim J., Jeong H., Kwon Y., Kim H., Lee S. (2014). Antiviral activity of hederasaponin B from Hedera helix against enterovirus 71 subgenotypes C3 and C4a. Biomol. Ther..

[63] Sarıkahya N.B., Kırmızıgül S. (2012). Antimicrobially active hederagenin glycosides from *Cephalaria elmaliensis*. Planta Medica.

[64] Ding R., Zhang Y., Xu X., Hou Y., Nie J., Deng X., Qiu J., Lv Q. (2022). Inhibitory effect of hederagenin on Streptococcus pneumoniae pneumolysin in vitro. Microbes Infect..

[65] Chakroborty A., Pritchard D.R., Bouillon M.E., Cervi A., Kraehenbuehl R., Wild C., Fenn C., Holdsworth P., Capner C., Padalino G. (2023). Modified hederagenin derivatives demonstrate ex vivo anthelmintic activity against fasciola hepatica. Pharmaceutics.

[66] Tian K., Su Y., Ding J., Wang D., Zhan Y., Li Y., Liang J., Lin X., Song F., Wang Z. (2020). Hederagenin protects mice against ovariectomy-induced bone loss by inhibiting RANKL-induced osteoclastogenesis and bone resorption. Life Sci..

[67] Santangelo R., Silvestrini A., Mancuso C. (2019). Ginsenosides, catechins, quercetin and gut microbiota: Current evidence of challenging interactions. Food Chem. Toxicol..

[68] Bai X., Duan Z., Deng J., Zhang Z., Fu R., Zhu C., Fan D. (2025). Ginsenoside Rh4 inhibits colorectal cancer via the modulation of gut microbiota-mediated bile acid metabolism. J. Adv. Res..

[69] Fang C., Yang H., Hui J., Fan D., Deng J. (2024). Natural bioactive compounds as potential sources for alleviation and treatment of androgenetic alopecia: A research advance. eFood.

[70] Han R.Y., Tan R.Z., Xu L.H., Lin J.Y., Li T., Su H.W., Li P., Liu P., Lan H.Y., Wang L. (2025). Activation of sclerostin inhibits Isg20-Mediated aerobic glycolysis ameliorating renal Fibrosis: The renoprotective mechanism of hederagenin in CKD. Redox Biol..

[71] Wang Y., Ai C., Wang H., Chen C., Teng H., Xiao J., Chen L. (2023). Emulsion and its application in the food field: An update review. eFood.

